# Effect of renin–angiotensin–aldosterone system inhibitor and statin medication on periodontal status of patients at risk of cardiovascular disease: a systematic review and meta-analysis

**DOI:** 10.1007/s44445-026-00135-1

**Published:** 2026-03-09

**Authors:** Zahid Affan Khalilurrahman, Saskia Kirana Anjani, Benso Sulijaya, Natalina Haerani, Robert Lessang, Nadhia Anindhita Harsas, Herlis Rahdewati

**Affiliations:** 1 Undergraduate Program, Faculty of Dentistry, Universitas Indonesia, Jakarta, Indonesia; 2 Departement of Periodontology, Faculty of Dentistry, Universitas Indonesia, Jakarta, Indonesia

**Keywords:** Renin–angiotensin–aldosterone system inhibitor, Angiotensin converting enzyme inhibitor, Angiotensin receptor blockers, Statin, Periodontal status

## Abstract

**Purpose:**

Among the various risks of cardiovascular disease, hypertension and hypercholesterolemia are the greatest risks. Renin–angiotensin–aldosterone system inhibitor medication, consisting of angiotensin converting enzyme inhibitors and angiotensin receptor blockers, as well as statins, is used to treat hypertension and hypercholesterolemia, respectively. The effect of this medication on inflammatory reactions has been demonstrated by various studies, as well as its effect on periodontal tissue. This study aims to determine the effect of renin–angiotensin–aldosterone system inhibitor medication and statins on the periodontal status of patients at risk of cardiovascular disease.

**Methods:**

Search for studies through electronic databases using the Preferred Reporting Items for Systematic Reviews and Meta-Analysis 2020 guidelines. Studies that met the inclusion criteria are included and assessed for risk of bias and then meta-analysed.

**Results:**

Qualitative synthesis showed inconsistent results regarding the effect of RAAS inhibitors on periodontal status. Meta-analysis between the statin group compared with other interventions such as diet found that the mean bleeding on probing index found a statistically significant difference with a mean difference of -13.4% (95% CI:[-23.08;-3.72], p = 0.007), the mean clinical attachment loss did not show any significant difference with a mean difference of -0.16 mm (95% CI: [-0.40;-0.07], p = 0.17), and mean probing depth there was a statistically significant difference between groups with a mean difference of -0.38 mm (95% CI:[-0.53;-0.23], p < 0.00001).

**Conclusion:**

The effect of renin–angiotensin–aldosterone system inhibitor medication in the form of angiotensin converting enzyme inhibitors and angiotensin receptor blockers in hypertensive patients on periodontal status showed inconsistent results and statin medication systemically could improve periodontal status, bleeding on probing index, and probing depth in patients with hypercholesterolemia.

**Supplementary Information:**

The online version contains supplementary material available at 10.1007/s44445-026-00135-1.

## Highlights

- ACEI and ARBs’ effect on periodontal status in hypertensive patients showed inconsistent results.
- Statin effect on periodontal status in hypercholesterolemia patients showed that it could improve the bleeding on probing index and the probing depth.

**Supplementary Information:**

The online version contains supplementary material available at 10.1007/s44445-026-00135-1.

## Introduction

Cardiovascular diseases represent a major group of diseases and remain the leading cause of mortality worldwide (Mensah et al. 2019; Roth et al. 2020). The World Heart Federation, in its latest report, World Health Report 2023, reported that the estimated number of deaths due to cardiovascular disease continues to increase and reached 18.6 million people in 2019 (Di Cesare et al. 2023). The most common cardiovascular diseases globally are ischemic heart disease/coronary heart disease, intracerebral hemorrhage, ischemic stroke, hypertensive heart disease, and so on (Vaduganathan et al. 2022). Some of the considerable risk factors for cardiovascular disease include increased blood pressure or hypertension, high levels of LDL cholesterol or hypercholesterolemia, obesity, smoking habits, and family history (Adhikary et al. 2022; Vaduganathan et al. 2022). According to the national survey data by the General Authority for Statistics in 2017, which included 24.012 households in Saudi Arabia, it was reported that the prevalence of hypertension was 9.2% among the Saudi population aged ≥ 15 years old, and the prevalence is paralleled with advancing age (≥ 65 years old) (Alenazi and Alqahtani 2023). Cardiovascular disease prevalence accounts 1.6% of the Saudi population aged 15 years and older, with the highest prevalence found in the age group of more than 65 years old (Alqahtani and Alenazi 2024). Hypercholesterolemia, reported in a cross-sectional survey by Al-Zahrani et al. 2021, showed 12.5% of the population had hypercholesterolemia and found that there was a positive association between increasing age and the prevalence of hypercholesterolemia (Al-Zahrani et al. 2021).

International clinical guidelines widely recommend that pharmacological management of cardiovascular diseases include renin-angiotensin system (RAS)-acting agents, beta-blockers, antithrombotic agents, and lipid-lowering drugs (Arnett et al. 2019). The drugs that are most commonly used as therapy for cardiovascular diseases are antihypertensive drugs such as angiotensin II receptor blockers (ARBs) and angiotensin-converting enzyme inhibitors (ACEI), which are included in the class of renin–angiotensin–aldosterone system (RAAS) inhibitors, as well as lipid-lowering agents such as statins (de Looper et al. 2017). Each group has its own function, for example, antihypertensive drugs for hypertension therapy and lipid-lowering to control cholesterol levels systemically (de Looper et al. 2017).

Periodontitis is one of the periodontal diseases that causes progressive damage to the periodontal tissue, resulting from inflammation of the periodontal tissue, with the main features of clinical attachment loss (CAL), radiographic loss of alveolar bones, the formation of periodontal pockets, and bleeding (Papapanou et al. 2018). The 2017 workshop by the American Academy of Periodontology (AAP) and European Federation of Periodontology (EFP) has classified periodontitis into 3 groups: necrotizing periodontal diseases, periodontitis as a manifestation of systemic diseases, and periodontitis. “Chronic” or “aggressive” periodontitis, which was known in the previous classification, is now grouped under periodontitis that are now characterized by the staging and grading system (Caton et al. 2018). Staging gives the information of the severity and complexity of the disease, while grading gives supplemental information about the progressivity of the disease (Caton et al. 2018).

Periodontitis has a high prevalence and morbidity, thus it constitutes a huge health problem. The prevalence of periodontal disease globally ranges from 40–50% (Nazir et al. 2020). The high severity of periodontitis can result in a person losing teeth, and further changes in mastication and aesthetic function can decrease a person's quality of life (Tonetti et al. 2017). Periodontitis itself has a variety of risk factors that can be divided into modifiable and non-modifiable factors. Modifiable risk factors include smoking habits, poor oral cavity hygiene, systemic diseases such as diabetes mellitus, and the use of certain medications (Alwithanani 2023; Lertpimonchai et al. 2017; M. A. Nazir 2017; Păunică et al. 2023). Non-modifiable factors of periodontitis are age and hereditary factors (Nazir 2017).

Cardiovascular disease has been linked to periodontitis because periodontal disease has pathogenic pathways similar to cardiovascular disease (Shetty et al. 2023). The relationship between the two can be explained through two mechanisms, namely the first mechanism, or direct pathway, with the invasion of periodontopathic pathogens into the blood vessels, and the second mechanism or indirect pathway through the release of inflammatory cytokines into the circulation of blood vessels, where these two mechanisms can result in the formation, maturation, and exacerbation of atheromatous plaques (Shetty et al. 2023). A consensus report by the World Heart Federation (WHF) and European Federation of Periodontology (EFP) states that patients with periodontitis may be at risk for endothelial dysfunction, coronary heart disease, and various other cardiovascular diseases (Sanz et al. 2020). Evidence suggests that periodontal treatment is associated with a reduced incidence of acute cardiovascular events and may influence the progression of cardiovascular disease (Herrera et al. 2023).

Hypertension and periodontitis are both conditions that can release pro-inflammatory cytokines from cells associated with the body's immune system, both of which can have an impact on cardiovascular disease (Del Pinto et al. 2020). Poorly controlled systemic inflammation can result in endothelial dysfunction following the development of hypertension (Khocht et al. 2017). Endothelial dysfunction can reduce periodontal vascularization and further interfere with the health of periodontal tissue (Khocht et al. 2017). These findings demonstrate a significant bidirectional relationship, where periodontitis is associated with an increased likelihood of hypertension, and vice versa (Takeguchi et al. 2021).

Another huge risk factor for cardiovascular disease, besides hypertension, is hypercholesterolemia, which has also been found to influence periodontal tissue (Khan and Mobin 2023). It was found that high cholesterol levels, or what is called hypercholesterolemia, have a positive relationship with periodontal disease (Khan and Mobin 2023; Watanabe and Cho 2014). High intake of cholesterol-containing foods has been associated with an increase in lipid profiles and subsequently an increased risk of periodontal disease, potentially through dyslipidemia and enhanced oral inflammatory responses (Khan and Mobin 2023). The relationship between the two can be explained through the systemic inflammatory system, which involves several pro-inflammatory cytokines, including IL-6, IL-1β, and so on (Gomes‐Filho et al. 2022).

The effect of the RAAS inhibitor group as an antihypertensive drug on the inflammatory process was found to be that the ACEI and ARBs groups could reduce inflammatory markers by suppressing the release of pro-inflammatory cytokines (Bryniarski et al. 2022; Silva et al. 2019). Benicky et al. reported that ARBs drugs were shown to inhibit pro-inflammatory cytokines secretion, namely TNF-α and IL-6, thereby suggesting a potential therapeutic role in inflammatory diseases (Benicky et al. 2009). In contrast to these findings, Rodrigues et al. reported that ACEI use was linked to a higher prevalence and greater severity of periodontitis (Rodrigues et al. 2016). Similarly, Kim et al. also reported that antihypertensive drugs can induce oral microbiota dysbiosis in periodontitis patients (Kim et al. 2023).

A systematic review study and meta-analysis by Bertl et al. examined the medication of lipid-lowering groups, namely statins show a positive effect on the treatment of periodontitis because, in addition to its effect on lowering blood cholesterol levels, it also has bone-promoting effects, anti-inflammatory, as well as antibacterial features. Evidence suggests that statins are capable of suppressing pro-inflammatory cytokines such as IL-1β, IL-6, and TNF-α (Rosenberg et al. 2019). Statins can promote osteoblast differentiation by upregulating bone morphogenetic protein-2 (BMP-2) and vascular endothelial growth factor (VEGF), thereby enhancing bone tissue formation (Balli et al. 2014; Dalcico et al. 2013). Several previous studies have demonstrated that the local application of statins as an adjunct to periodontal therapy has a beneficial effect on periodontal parameters (Greethurst et al. 2024a; Petit et al. 2019a; Sinjab et al. 2017). Contrary to other studies, there is a study by Kwon et al., which states that systemic statin use increases the risk of developing periodontitis by 1.32 times (Kwon et al. 2022).

The use of these drugs in treatment for hypertension and hypercholesterolemia globally continues to increase over time, with the most used groups including antihypertensive drugs in the form of RAAS inhibitors and lipid-lowering agents such as statins (Yan et al. 2021). Various studies examined the effects of several antihypertensive drug groups, such as ACEI and ARBs, as well as lipid-lowering agents, such as statins, on periodontal status (Bertl et al. 2018; Muniz et al. 2018; Petit et al. 2019b). However, the studies showed different results. Therefore, this systematic review and meta-analysis aim to determine the effect of RAAS inhibitors and statins on the periodontal status of patients at risk of cardiovascular disease, specifically patients with hypertension or hypercholesterolemia.

## Materials and methods

A retrospective observational systematic review and meta-analysis were performed in accordance with the PRISMA 2020 guidelines. Systematic review and Meta-analysis enable the statistical combination of findings from multiple similar studies to generate comprehensive qualitative and quantitative evidence, respectively. The study protocol is registered in the PROSPERO database under the registration number CRD42024583905.

### Inclusion and exclusion criteria

The inclusion criteria are: studies regarding the effect of ACEI medication, ARBs, or statins on the periodontal status of patients at risk of cardiovascular disease in the form of hypertension or hypercholesterolemia; publications in English; publications in the last 10 years; studies with the main outputs are periodontal status in the form of periodontal index (bleeding on probing index, plaque index, and gingiva index), clinical attachment loss, and probing depth; systemic administration of ACEIs, ARBs, or statins; and study models of clinical studies, clinical trials, and randomized controlled trials. Exclusion criteria are as follows: review study models and participants with other systemic diseases.

### Search strategy

The researcher conducted a study search with the PRISMA 2020 guidelines on four electronic databases, namely PubMed (https://pubmed.ncbi.nlm.nih.gov/), ProQuest (https://www.proquest.com/), EBSCO (https://www.ebsco.com), and Springerlink (https://link.springer.com/). The research question in this study is "Is there an effect of renin–angiotensin–aldosterone system inhibitor and statin medication on periodontal status of patients at risk of cardiovascular disease?". The research question was formulated using the PICO framework, which comprises Population, Intervention, Comparison, and Outcome. The population (P) included in this study is patients with hypertension or hypercholesterolemia. The interventions (I) are ACEI, ARBs, or statins. The comparison (C) is another medicated comparison. Lastly, the outcome (O) is primary outputs of periodontal status in the form of periodontal index (BOP index, plaque index, and gingiva index), loss of clinical attachment, and probing depth; and secondary outputs in the form of diagnosis of periodontal disease.

Based on the PICO framework, relevant keywords were identified and combined, including renin–angiotensin–aldosterone system inhibitors, angiotensin-converting enzyme inhibitors, angiotensin receptor blockers, statins, periodontal status, periodontal parameters, and periodontitis. The search strategy employed Boolean operators (“OR” and “AND”) to construct combinations of these keywords, full search strategy can be seen in supplementary materials 1.

### Selection of the studies

The electronic search was conducted across PubMed, ProQuest, EBSCO, and SpringerLink using predefined keywords. All retrieved records were imported into the Rayyan web-based platform for study selection. Duplicate studies identified across databases were removed. Screening was initially performed based on titles and abstracts to exclude irrelevant articles. Full-text assessment of the remaining studies was then conducted to determine eligibility according to the predefined inclusion and exclusion criteria, in accordance with PRISMA 2020 guidelines. The selection of the studies was performed by two reviewers blinded. No disagreements occurred during the selection process.

### Risk of bias assessment and analysis

All included studies were assessed for risk of bias using the Cochrane Risk of Bias in Non-randomized Studies of Interventions (ROBINS-I) tool for non-randomized controlled trials (Sterne et al. 2016). Data synthesis was conducted both qualitatively and quantitatively using Review Manager (RevMan) software. The pooled effect estimates were calculated, and the results were presented as an overall mean with corresponding forest plots.

## Results

### Research identification and selection

**Fig. 1:**
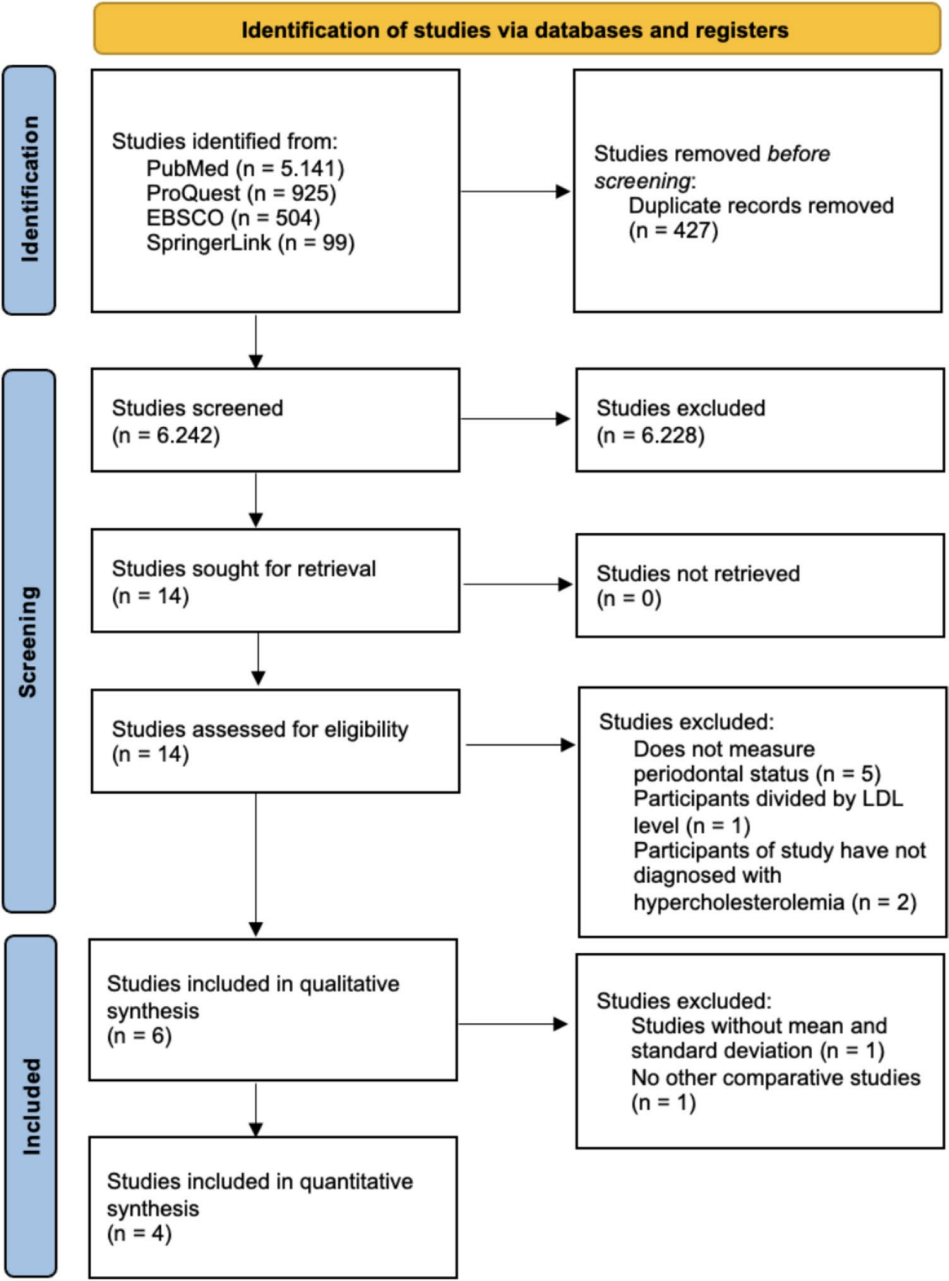
Diagram of the identification process of the study according to the Flow of PRISMA 2020

The electronic database search yielded a total of 6,669 records, including 5,141 from PubMed, 925 from ProQuest, 504 from EBSCO, and 99 from SpringerLink. After the removal of 427 duplicate records using the Rayyan platform, 6,242 studies remained for screening. Title screening excluded 6,226 records that did not meet the predefined inclusion and exclusion criteria. Abstract screening was subsequently conducted on 16 studies, resulting in the exclusion of two additional articles. Fourteen full-text articles were then assessed for eligibility. Of these, five studies did not report periodontal status, one study compared groups based on LDL levels, and two studies did not include patients diagnosed with hypercholesterolemia. Consequently, six studies were included in the qualitative synthesis. One study was excluded from quantitative synthesis due to the absence of mean and standard deviation data, and another study lacked a comparative group. The Rayyan platform was used for duplicate removal and solely as a screening management tool; final inclusion and exclusion decisions were made by the reviewers. Therefore, quantitative synthesis (meta-analysis) was performed on four studies (Fig. 1).

### Risk of bias assessment

**Fig. 2:**
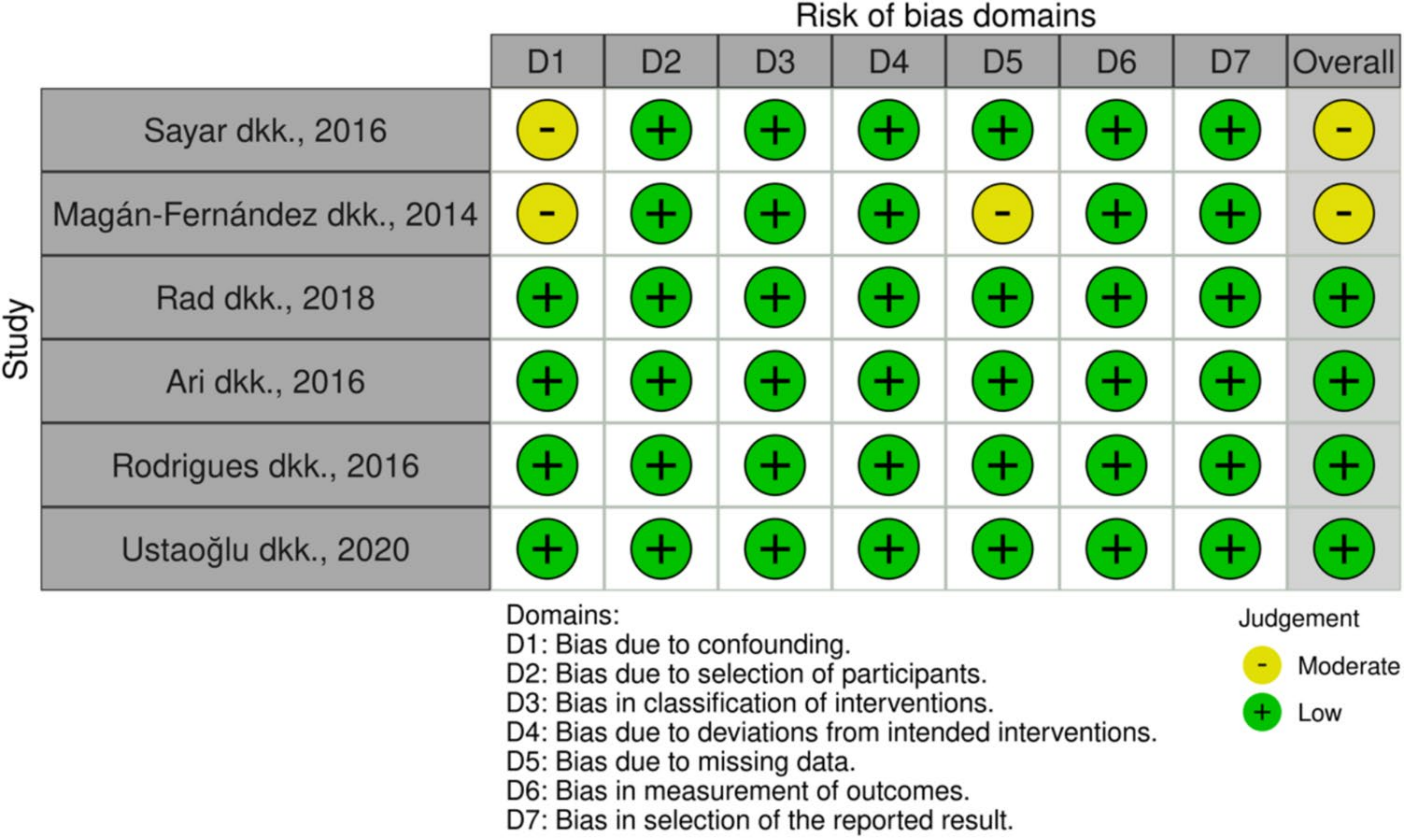
Risk of bias assessment for non-randomized clinical trials

The risk of bias assessment was carried out by 2 groups with each consisting of 2 assessors, who then discussed the results of their respective bias risk assessments until an agreement was reached. The risk of bias assessment results are as follows: four studies had a low risk of bias, and two studies had a medium risk of bias. The results of the bias risk assessment can be seen in Fig. 2.

### Qualitative synthesis

Qualitative synthesis was performed on six included studies by extracting key information from each article (Ari et al. 2016; Magán-Fernández et al. 2014; Rad et al. 2018; Rodrigues et al. 2016; Sayar et al. 2016; Ustaoğlu et al. 2020). The extracted data included the authors’ names, year of publication, study design, type and duration of systemic medication use, number of participants, periodontal status parameters (bleeding on probing, plaque index, gingival index, clinical attachment loss, and probing depth), periodontal disease characteristics, and study outcomes. All of the studies have no marginal bone loss data. Periodontal disease given in all the included studies did not adhere to the 2017 Workshop of Periodontal and Peri-Implant Diseases and Conditions. These data are summarized in Table 1.

**Table 1:** Qualitative synthesis results

Author (Year)	Study model	Systemic medication/duration of consumption	Number of participants	Periodontal Status			Periodontal Disease	Result
				Periodontal index	Clinical attachment loss (mm)	Probing depth (mm)		
(Sayar et al. 2016)	Cross sectional	Statins/> 3 months	150 Statin (n = 50) Diet (n = 50) Normolipidemic (n = 50)	BOP: 62% ± 49 PI: 2,32 ± 0,54	1,83 ± 0,67	2,76 ± 0,39	ND	**Primary outcomes:** There was a significantly lower difference in mean of clinical attachment loss and probing depth between the statin group and the diet group
(Magán‐Fernández et al., 2014)	Cross sectional	Statin (Simvastatin)/> 3 months	73 Statin (n = 29) Diet (n = 28) Normolipidemic (n = 16)	BOP: ND PI: 39% ± 34	2,7 ± 1,2	2,0 ± 0,9	ND	**Primary outcomes:** There was no significant mean difference in all measurements of periodontal status between the statin group and the diet group or normolipidemic group
(Rad et al. 2018)	Non randomized controlled trial	Statin (Simvastatin dan Lovastatin)/3 months	40 Statin (n = 20) Normolipidemic (n = 20)	BOP: 11,52 ± 34,50 GI: 0,605 ± 1,95 PI: 0,696 ± 1,8	0,510 ± 2,95	0,500 ± 3,75	Chronic periodontitis	**Primary outcomes:** 1. There was a significant difference between the statin and normolipidemic groups in the measurement of gingival index 2. There was no significance from other periodontal status measurements between the statin group and the normolipidemic group
(Ari et al. 2016)	Cross sectional	Statin/ND	127 Statin (n = 53) Diet (n = 26) Normolipidemic (n = 48)	BOP Healthy: 0% Gingivitis: 76,47 ± 20,67% Periodontitis: 73,86 ± 24,97% GI Healthy: 0,26 ± 0,33 Gingivitis: 1,03 ± 0,33 Periodontitis: 1,39 ± 0,68 PI Healthy: 0,29 ± 0,31 Gingivitis: 0,70 ± 0,40 Periodontitis: 1,02 ± 0,69	Healthy: 1,33 ± 0,21 Gingivitis: 1,19 ± 0,64 Periodontitis: 3,58 ± 0,99	Healthy: 1,27 ± 0,17 Gingivitis: 1,31 ± 0,36 Periodontitis: 2,30 ± 0,85	Gingivitis and Chronic Periodontitis	**Primary outcomes:** 1. The BOP value in the gingivitis group in the statin group was significantly smaller than in the normolipidemic group 2. PI and GI values in the healthy periodontal group in the statin group were significantly higher than in the normolipidemic group 3. There was no significant difference in the measurement of clinical attachment loss status in each group 4. The value of probing depth in the periodontitis group in the statin group was significantly smaller than in the normolipidemic group
(Rodrigues et al., 2016b)	Case–control	ACEI/ND	65 ACEI (n = 30) Non-ACEI (n = 35)	BOP: 60,2% (22,2–100) PI: 75,1% (10,7–100)	2,69 (1,98–4,99)	3,01 (2,24–5,25)	Chronic Periodontitis	**Primary outcomes:** There were significant differences in periodontal status that were more severe in the ACEI group than in the non-ACEI group **Secondary outcomes:** ACEI Medication also had a 3.2-fold greater risk of having sites with a probing depth of ≥ 5 mm and a threefold greater risk of having sites with clinical attachment loss of ≥ 5 mm
(Ustaoğlu et al. 2020)	Cross sectional	ACEI/≥ 2 years ARB/≥ 2 years CCB/≥ 2 years	131 ACEI (n = 40) ARB (n = 40) CCB(n = 51)	BOP ACEI: 50% (29,6) ARB: 50% (35,9) GI ACEI: 1,53 (0,3) ARB: 1,38 (0,2) PI ACEI: 2,0 (1,0) ARB: 2,0 (0,6)	ACEI: 2,7 ± 0,5 ARB: 2,56 ± 0,6	ACEI: 2,58 ± 0,5 ARB: 2,39 ± 0,6	Drug induced gingival overgrowth	**Primary outcomes:** There was a significant difference in the GI index measurement of the ARB group compared to the CCB

“Bleeding on probing” = BOP; “Gingival index” = GI; “Plaque index” = PI; “angiotensin-converting enzyme inhibitor” = ACEI; “angiotensin receptor blockers” = ARB; “calcium channel blockers” = CCB; “No Data” = ND

Based on the qualitative synthesis, the effect of RAAS Inhibitor medication in the form of ACEI and ARB on the periodontal status of patients with hypertension showed inconsistent results and there were only 2 studies that were synthesized so the effect could not be determined (Rodrigues et al. 2016; Ustaoğlu et al. 2020).

### Quantitative synthesis

Quantitative synthesis or meta-analysis was carried out in four studies using Mean difference analysis from RevMan 5.4. software with a Confidence Interval (CI) of 95%, then a p-value was determined to determine the statistically significant difference.

**Fig. 3:**
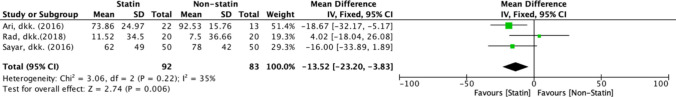
Forest-plot measurement of bleeding on probing index between statin medication and other interventions

**Fig. 4:**
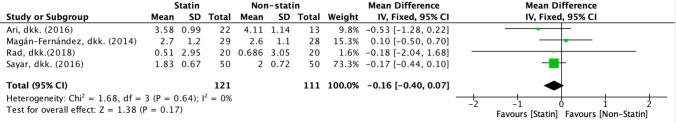
Forest-plot measurement of clinical attachment loss between statin medication and other interventions

**Fig. 5:**
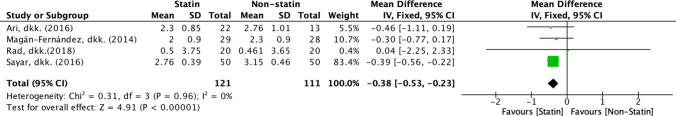
Forest-plot probing depth measurement between statin medication and other intervention

Studies on the effect of statin medication on periodontal status in the form of bleeding on probing index, clinical attachment loss, and probing depth have been collected and synthesized quantitatively as provided in Figs. 3, 4, 5. The plaque index and gingival index were not quantitatively analyzed because the indices used by each study were different. Studies on the effects of RAAS Inhibitors were not quantitatively synthesized because one study did not have a mean value and standard deviation so that the other studies did not have a comparative study. Quantitative synthesis of bleeding on probing index was carried out with three studies while clinical attachment loss and probing depth were carried out with four studies. A meta-analysis was performed to compare the mean difference between periodontal index, clinical attachment loss, and probing depth of the hypercholesterolemia patient group with statin medication compared to other interventions, such as diet.

From the results of data synthesis, it was found that the average bleeding on probing index was found to be statistically significant with an average difference of −13.4% (95% CI:[−23.08;−3.72], p = 0.007) (Fig. 3). Measurements of mean clinical attachment loss did not show any significant difference between statin and non-statin groups (Fig. 4). Therefore, there is no effect of statin medication on clinical attachment loss in patients with hypercholesterolemia. In the measurement of the mean depth of probing, there was a statistically significant difference between groups with an average difference of −0.38 mm (95% CI:[−0.53;−0.23], p < 0.00001) (Fig. 5). Therefore, there is an effect of statin medication on the depth of probing in patients with hypercholesterolemia.

## Discussion

### Effect of RAAS inhibitor on periodontal status in hypertensive patients

The two studies that discussed the medication of RAAS Inhibitor on periodontal status in hypertensive patients, namely the study by Rodrigues et al. (2016) and Ustaoğlu et al. (2020) had a low risk of bias assessment in all domains. A study by Rodrigues et al. (2016) examined the effect of ACEI medication on periodontal status in patients with controlled hypertension and chronic periodontitis by comparing the test group taking ACEI and the control group taking medications other than ACEI with 70% of the samples in the control group using ARBs (Rodrigues et al. 2016). Both groups took antihypertensive drugs in combination with diuretics, adrenergic inhibitors, or CCB. The second study by Ustaoğlu et al. (2020) compared the effects of ACEI, ARBs, and CCB medications on the risk of experiencing drug-induced gingival overgrowth and its periodontal status (Ustaoğlu et al. 2020).

A study by Rodrigues et al. (2016) showed that all periodontal statuses in the form of BOP, PI, clinical attachment loss, and probing depth showed that there was a significant difference (p < 0.05) between the case group and the control group, with the case group measurement value showing a larger number than the control group (Rodrigues et al. 2016). However, the differences in probing depth and clinical attachment loss between the case and control groups were only 0.47 mm and 0.26 mm, respectively, indicating that these changes were not clinically significant. The results of the study by Ustaoğlu et al. (2020) showed different results, namely, ACEI medication compared to other antihypertensive medications did not show any significant difference in all periodontal status measurements. However, the GI measurement value in the ARBs medication group was significantly smaller than in the CCB medication group, but the difference was only 0.4 points, with the GI value of ARBs group being 1.38 and the CCB group being 1.78 thus, it did not have a clinically significant effect (Ustaoğlu et al. 2020).

Inconsistent results between the two studies are hypothesized due to some differences between these two studies, the first being the length of treatment duration in the samples used in both studies. The study by Rodrigues et al. (2016) had an average duration of antihypertensive drug medication of 7.5 years for the ACEI group and for 6.1 years for the non-ACEI group, while in the study by Ustaoğlu et al. (2020) the duration of medication was shorter, precisely 3 years for the ACEI and CCB groups and 3.5 years for the ARBs group. The second difference is the therapeutic approach, which in the study by Rodrigues et al. (2016) both groups used antihypertensive drug therapy in combination with other antihypertensive groups (combination therapy) such as diuretics, adrenergic inhibitors, or CCB in both groups while in the study by Ustaoğlu et al. (2020) all three groups only used single-drug therapy. The third difference is the difference in the type of study used, which in the study by Rodrigues et al. (2016) using a case–control study and Ustaoğlu et al. (2020) using a cross-sectional study model.

Previous research, a retrospective cohort study by Chatzopoulos et al. (2023) examined the prevalence of systemic medication consumption among patients with periodontitis, which was defined as stage III and stage IV periodontitis, and patients with healthy periodontal (Chatzopoulos et al. 2023). The study stated that patients with periodontitis have a higher consumption of ACEI medication compared to patients with healthy periodontal tissues (Chatzopoulos et al. 2023). Of the five types of ACEIs used in this study, benazepril, quinapril, ramipril, enalapril, and lisinopril, there were three types of ACEIs that were statistically significant in the periodontitis patient group, particularly ramipril, enalapril, and lisinopril (Chatzopoulos et al. 2023). The study also further examined the multivariable regression between the relationship between systemic medication and periodontal tissue conditions, which found there is a 4.847 times greater risk of developing periodontitis by consuming ACEI (Chatzopoulos et al. 2023). The same results were also shown by a case–control study by Wang et al. (2020), namely in elderly patients, the consumption of ACEI medication was significantly more consumed in the group of patients suffering from periodontitis and in the regression test it also showed that there was a 1.64 times greater risk of suffering from periodontitis if consuming ACEI drugs class (Wang et al. 2020). Regarding the use of ARBs medication, a study by Pająk-Łysek et al. (2021) stated that there was a strong association between ARBs medication and periodontitis, which was 3.32 times the greater risk after controlling for all confounding factors (Pająk-Łysek et al. 2021).

These findings may be explained by the potential of angiotensin-converting enzyme inhibitors (ACEIs) to enhance the pro-inflammatory properties of periodontal pathogens. Evidence suggests that adverse effects of ACEIs, such as cough and angioedema, are associated with increased kinin activity resulting from angiotensin-converting enzyme inhibition; kinin is a known pro-inflammatory mediator that also participates in the inflammatory processes of periodontal disease (Souza et al. 2019). Furthermore, ACEI use may potentiate T helper 1 (Th1) polarization by increasing interleukin-12 (IL-12) production through the activation of kinin B2 receptors expressed on dendritic cells (Rodrigues et al. 2016). In addition, reduced salivary flow may contribute to the association between angiotensin receptor blocker (ARB) use and periodontitis, as hyposalivation is a recognized adverse effect of ARB therapy and may increase susceptibility to periodontal disease (Pająk-Łysek et al. 2021).

Thus, the medication of ACEIs and ARBs can increase the risk of exacerbation of periodontal status and the risk of periodontal diseases such as periodontitis. However, differences in the results of measuring periodontal status between the RAAS Inhibitors such as ACEI and ARBs or with other antihypertensive classes showed inconsistent results. Therefore, further research is needed by considering the duration of treatment and also the use of single-drug therapy so that there are no confounding factors from other antihypertensive drugs.

### Effect of statins on periodontal status in hypercholesterolemia patients

Of the four studies that discussed the effect of statin use on the periodontal status of patients with hypercholesterolemia, two studies had a low bias risk assessment and two studies had a moderate bias risk assessment, which are studies by Sayar et al. (2016) and Magán-Fernández et al. (2014). Both studies had a moderate bias risk value due to the confounding bias domain because both studies had a risk of confounding factors in the samples used, but in both studies, the factors were controlled. In the study of Magán-Fernández et al. (2014), the domain bias due to missing data was also considered moderate because there was data in the missing sample, but it had no effect on the data analysis.

Three of the four studies (Sayar et al. (2016); Rad et al. (2018); Ari et al. (2016)) regarding the effect of statins on periodontal status in hypercholesterolemia patients showed that there was a significant difference in the average value of periodontal status measurement between the statin group and the diet group or normolipidemic group (Ari et al. 2016; Rad et al. 2018; Sayar et al. 2016). The measurement of bleeding on probing index was only significant in a study by Ari et al. (2016), where the BOP index value in the statin group was significantly smaller than the normolipidemic group in the group with gingivitis (Ari et al. 2016). GI measurements showed similar results between studies by Rad et al. (2018) and Ari et al. (2016) where both showed significantly higher results compared to the normolipidemic group in the group with healthy periodontal tissue conditions (Ari et al. 2016; Rad et al. 2018). The PI measurement was proven to be significant by two studies, Sayar et al. (2016) and Ari et al. (2016), where both showed a lower significant difference in the statin group compared to the normolipidemic group in the group with healthy periodontal tissue conditions and also compared to the diet group in the study by Sayar et al. (2016). (Ari et al. 2016; Sayar et al. 2016).

Clinical attachment loss showed a statistically significant difference in only one study, namely Sayar et al. (2016), which reported a clinically meaningful lower value in the statin group compared with the diet group (Sayar et al. 2016). The mean value of probing depth showed a significant difference in the study by Sayar et al. (2016) and Ari et al. (2016). In the study by Sayar et al. (2016), the mean probing depth was significantly lower in the statin group compared with the diet group, but no significant difference was observed when compared with the normolipidemic group. Conversely, Ari et al. (2016) reported a significantly lower mean probing depth in the statin group compared with the normolipidemic group in patients with periodontitis, whereas no significant difference was found when compared with the diet group (Ari et al. 2016; Sayar et al. 2016).

One study by Magán-Fernández et al. (2014) showed no significant difference in the mean measurement value of the entire periodontal status (Magán-Fernández et al. 2014). Although there was no significant difference in the mean value of the measurement of periodontal status, the study further performed a multivariate regression test that showed a reduction of clinical attachment loss of 0.8 mm in the statin group compared to the control or normolipidemic group (Magán-Fernández et al. 2014). Other outcomes in this study also tested the association between periodontal status and lipid profile levels, which also showed no significant relationship between the two (Magán-Fernández et al. 2014).

Based on the meta-analysis conducted in this study, only two parameters produced significant values; the first was the bleeding on probing index, with p = 0.007, with an average BOP index value reduced by 13.40% in the statin group compared to the non-statin group. The second periodontal status was the probing depth, with p < 0.00001, with the average probing depth value reduced by 0.38 mm in the statin group compared to the non-statin group which although statistically significant but not clinically significant. By the time this article is written, there has not been any universally established minimum clinically important difference value for periodontal probing depth and bleeding on probing index. However, to our understanding, a decrease of probing depth by 1 mm might be clinically significant for some cases. For instance, the decrease of probing depth by 1 mm from 4 to 3 mm can be significant since the probing depth of 4 mm is considered a pathologic condition and 3 mm is considered a healthy periodontal condition according to the 2017 classification of periodontal disease (Chapple et al. 2018). While on other cases, for instance, a 10 mm probing depth decreased by 1 mm to 9 mm, both conditions are still considered as pathological conditions. Regardless of the probing depth value, the BOP index, which was reduced by 13.40% has a high impact on periodontal health.

Muniz et al. (2018) in their systematic review and meta-analysis study examined the effect of statins as adjuvant therapy on non-surgical periodontal therapy (Muniz et al. 2018). In the study, three types of statin administration were examined, which are by systemic, dentifrice, and local. Of the statins administered systemically, only one study showed an improvement or improvement in probing depth measurements, while the other two studies showed no significant changes (Muniz et al. 2018). There has only been one study of statins administered via dentifrice, but it has been shown that atorvastatin can improve clinical adhesion, reduce probing depth, and bleeding on probing (Muniz et al. 2018). Meta-analysis tests were performed on locally administered statins and showed that after 6 months, there was a reduction in probing depth of 1.93 mm and an increase in clinical adhesion by 1.82 mm, but in this study, the heterogeneity of the data used was high (Muniz et al. 2018). A similar study by Greethurst et al. (2024a, b) examining the local use of statins as adjunct therapy in non-surgical periodontal care also showed significant improvements in periodontal status, such as probing depth, clinical attachment loss, infrabony defect, and radiographic bone loss (Greethurst et al. 2024b). Both studies showed similar results to this study, which showed an improvement in periodontal status. However, there are a few differences with this study, which are: the statin administration methods used, the use of statin as an adjunctive agent after periodontal treatment not as a therapy for hypercholesterolemia and using systemically healthy samples while in this study used patients with hypercholesterolemia (Greethurst et al. 2024b; Muniz et al. 2018).

The findings of the present study may be explained by evidence from previous research demonstrating that statins have a significant anti-inflammatory effect by reducing levels of pro-inflammatory markers, including IL-8, IL-6, TNF-α, and IL-1β (Kavalipati et al. 2015). In addition, statins have demonstrated in vitro antibacterial activity against both Gram-positive and Gram-negative bacteria (Masadeh et al. 2012). In a study by Dalcico et al. (2013) simvastatin reduced inducible nitric oxide synthase (iNOS) levels, RANK, RANKL, and increased OPG levels in periodontal tissue (Dalcico et al. 2013b). Consistent with these findings, Tabrizi et al. (2019) in their systematic review and meta-analysis study showed that systemic statin therapy significantly decreased inflammatory markers, including C-reactive protein (CRP), TNF-α, IL-6, and IL-1 (Tabrizi et al. 2019).

## Conclusions

Based on the analysis, the effects of renin–angiotensin–aldosterone system inhibitor therapy—specifically angiotensin-converting enzyme inhibitors and angiotensin receptor blockers—on periodontal status in hypertensive patients were found to be inconsistent. In contrast, systemic statin therapy was associated with improvements in periodontal parameters, particularly bleeding on probing and probing depth, in patients with hypercholesterolemia.

## Supplementary Information

Below is the link to the electronic supplementary material.

Supplementary file1 (DOCX 16 KB)

## Data Availability

All data generated or analysed during this study are included in this article.
