# Supplementary material for: Effect of renin–angiotensin–aldosterone system inhibitor and statin medication on periodontal status of patients at risk of cardiovascular disease: a systematic review and meta-analysis
Source: Saudi Dent J. 2026 Mar 9;38(3):27. doi: 10.1007/s44445-026-00135-1 (PMC12972301; doi:10.1007/s44445-026-00135-1)
Supplement: Supplementary file 1 — Supplementary file1 (DOCX 16 KB) [file 44445_2026_135_MOESM1_ESM.docx]

**Supplementary material**

Article title : Effect of Renin-Angiotensin-Aldosterone System Inhibitor and Statin Medication on Periodontal Status of Patients at Risk of Cardiovascular Disease: A Systematic Review and Meta-Analysis

Journal Name : The Saudi Dental Journal

Author names :

- Zahid Affan Khalilurrahman^1^

- Saskia Kirana Anjani^1^

- Benso Sulijaya^2^

- Natalina^2^

- Robert Lessang^2^

- Nadia Anindhita Harsas^2^

- Herlis Rahdewati^2^

Affiliation :

^1^Undergraduate Program, Faculty of Dentistry, Universitas Indonesia, Jakarta, Indonesia

^2^Departement of Periodontology, Faculty of Dentistry, Universitas Indonesia, Jakarta, Indonesia

Corresponding author :

Herlis Rahdewati
Email: herlis.rahdewati02@ui.ac.id

Table 1. Full Search Strategy

| *Database* | Complete Search Strategy | Total |
| --- | --- | --- |
| PubMed | ((((((periodontal status) OR (periodontal parameter)) OR (periodontal disease)) AND (renin-angiotensin-aldosterone system inhibitor)) OR (angiotensin-converting enzyme inhibitor)) OR (angiotensin receptor blockers)) OR (statin)  Filters: Clinical Study, Clinical Trial, Randomized Controlled Trial, in the last 10 years | 5.141 |
| ProQuest | (periodontal status OR periodontal parameter OR periodontal disease) AND (*renin-angiotensin-aldosterone system inhibitor* OR angiotensin-converting enzyme inhibitor OR angiotensin receptor blockers OR statin)  Additional limits - Date: From 2014 to 2024; Source type: Scholarly Journals; Language: English | 925 |
| EBSCO | periodontal status OR periodontal parameters OR periodontal disease AND renin-angiotensin-aldosterone system inhibitor OR angiotensin converting enzyme inhibitor OR angiotensin receptor blockers OR statin  Date of Publication: 2014-2024; English | 504 |
| SpringerLink | ("periodontal status" OR "periodontal parameter" OR "periodontal disease") AND ("renin-angiotensin-aldosterone system inhibitor" OR "angiotensin-converting enzyme inhibitor" OR "angiotensin receptor blockers" OR "statin") | 99 |
